# Atlanta Society of Medicine

**Published:** 1889-04

**Authors:** 


### Society Reports.

### ATLANTA SOCIETY OF MEDICINE.

MEETING March 5, 1889.

President Hunter P. Cooper, M. D., in the chair.

### UREAMIA FOLLOWING CONFINEMENT.

Dr. Noble reported a case of labor, to which he was called in consultation with Dr. Woodward—said, that on examination, he found an arm and shoulder presenting—turned, and brought the feet down—the hands remaining alongside of the head (a very large one), passed up a blunt hook and brought the arms down also—he then rotated the head, bringing the occiput into hollow of the sacrum—induced acute flexion, by placing his finger into the mouth, and delivered a dead baby, which weighed eighteen pounds. He tried to resuscitate the infant, but failed.

The patient did not have good attention, and on his first visit after the accouchment, found her in a filthy bed—temperature 106 degrees—pulse 140. No lochial discharge—no urine in the bladder--diagnosed ureamia—washed out the uterus with carbolized water, and ordered opium, ½ grain, and sulphate quinine, 5 grains, together with tincture digitalis, 15 drops—every three hours. At his next visit, found her in a perspiration, with better pulse; continued opium and quinine, in same doses, but increased the digitalis to 18 drops.

At next visit he noticed that the pulse had fallen to 120, and temperature to 104¾, and that there was urine in the bladder—this was 10 P. M. of the second night of his attendance. Next morning, pulse 100, temperature 102. From this, the patient made an excellent recovery.

Dr. Noble said that he believed the digitalis to be the prime factor in relieving the ureamia.

PELVIC ABSCESS.

Dr. Noble reported two cases of pelvic abscess—the one, a young lady, the abscess was the result of pelvic inflammation, consequent upon catarrhal fever. He opened the abscess in front of the uterus and a pint and a half of pus was discharged—put in drainage tube—the patient did well. The other case, the cause was constipation; abdomen was dull up to umbilicus, and there was fluctuation. Applied cocaine to the vaginal wall immediately behind the os uteri, nicked the mucous membrane, introduced a meatrotome, and cut from one side to the other, then introduced finger, and pus came away to the amount of one quart; drainage tube put in, and the usual treatment instituted—washing of carbolized water, etc. Said that this patient had suffered from constipation for some time. Large masses of fœcal matter had to be removed, together with chalky masses, as large as an egg, from the rectum.

EXPLORATORY LAPAROTOMY.

Dr. Hardon reported a case of negro woman, aged 38 years, who had a fibroid tumor of the uterus, which had developed some five years ago. Since then, it had gradually increased, and at the time of his visit it was the size of a fœtal head; no serious trouble until shortly before sending for him, when she suffered from recurrent peritonitis. On examination he found tenderness of the abdomen, high pulse, and high temperature, with severe pain, which required morphia continuously. He learned that she had been treated with ergot, without apparent effect; her suffering being so acute, and seeing nothing else to do, he made an exploratory incision, found in the abdominal cavity a collection of ascitic fluid, which he allowed to escape through the incision; found that the tumor lay embedded in a plastic exudation which extended all over the pelvic organs, and which bound tumor and organs to the pelvis in such a manner as to prevent separating the mass without great danger and difficulty. Said he concluded to let it alone, and emptying abdomen of the fluid, closed the incision.

The patient rapidly improved, began to gain strength, and appetite. Six months after the operation she had gained thirty pounds, walked a long distance, and was able to do her own work, and has had no attack of peritonitis since.

Dr. Hardon said the operation was negative, so far as the removal of the mass was concerned, but that the effect of the operation was the improvement of the woman's health. Dr. Hardon said there were on record, many cases of chronic peritonitis, causing accumulation of fluid in the abdomenal cavity, that had been cured by exploratory incision. He had seen where tubercular peritonitis had been thus apparently cured by this means; in fact, he believed it was now the adopted treatment for this condition, and mentioned such authority as Lawson Tail, and Gill Wylie, in support of his statement.

Dr. Noble remarked that he remembered a case of peritonitis recurring at each period of menstruation ; the abdomen was opened, the pelvic organs were bound down by plastic exudation; no fluid. Patient improved very much, there was less pain at periods, and her general health was better. A year or so, after the first operation, another was performed, at request of patient; no fluid was found; yet the patient improved still more, and was now about well.

### ACUTE PROLAPSUS UTERI.

Dr. Duncan reported a case of lady, married three months; when called to see her, learned that she had had but one period after marriage, and some morning sickness. He found her suffering great pain, used morphia hypodermically, then deodorized tinct. of opium, and tinct. of gelseminum, every two hours; made examination, found uterus large and fixed in the pelvis; applied tanpons of glycerine and cotton for two days, with the result of relieving the pains, and of elevating the uterus to a normal position. Dr. Duncan gave the credit to the glycerine and cotton.

### LACERATION OF EYE-BALL.—FRACTURE OF ZYGOMATIC ARCH, SUPRA-ORBITAL ARCH, MALAR BONE, AND VAULT OF ORBIT.

Dr. Duncan reported for Dr. Divine a case of accident. Mr. A. M. T., aged 54 years, thrown from his wagon, sustained following injuries: Laceration of the right eye, and fracture of the surrounding bony structures, (z. a., s. a., m. b., and v. of o.). Dr. Divine removed the lacerated eye-ball, and a few spiculæ of bone, also a small piece of wood, which had been driven into the vault of orbit. The torn parts were brought together with continuous suture of iron-dyed silk. Fractures were thoroughly adjusted, (being but slight displacements, this was easily accomplished). Antiseptics freely applied, and a bichloride bandage over all, and a mercurial purge ordered. Morphia was given occasionally for the relief of pain, for the first two days. Redressed the wound on the fifth day; no suppuration; wound had healed by first intention. The eighth day, all stitches were removed, and the patient regarded as nearly well. Dr. Duncan said that the patient's temperature was never higher than 100 degrees, nor his pulse more than 96.

### ANÆSTHESIA OF INFRA-ORBITAL NERVE.

Dr. Nicolson reported case of a man who had stepped from a moving engine, and in falling had slid over several cross-ties, receiving a gash on the left side of forehead, extending across left eye, down to left cheek, and a severe contusion of left eye. Patient recovered quickly. Sometime after, he returned to him, and complained of loss of sensation in the lower lid of the injured eye, and surrounding parts. Dr. Nicolson said, on examination, he found quite a projection of the inferior orbital ridge, just below the inner canthus of the eye, and concluded that the force of the blow had been sustained at this point, causing a detachment of a small piece of the ridge, and driving it forward, which interfered with the functions of the infra-orbital nerve, and was the cause of the complete anæsthesia of the parts.

### DELAYED LABOR, OCCASIONED BY RIGIDITY OF THE OS UTERI.

Dr. Johnson reported a case of delayed labor, with rigid os.

Said the woman at first had very strong pains, which had no effect upon advancing the labor. Gave morphia, and waited; after a few hours the pains ceased entirely; he then, (presentation being normal,) concluded to use the forceps, ruptured the membrane, and introduced one blade. As he did so, the pains came on with renewed vigor, he withdrew the blade, and sent for Dr. McRae to assist him. On Dr. McRae's arrival, chloroform was administered, and Dr. McRae introduced the forceps, and slowly delivered the woman. Dr. McRae said the peculiarity of the case was a rigid condition of the os, and the fact of the introduction of the forceps bringing on powerful expulsive pains. That they were so strong at one time that he had to withdraw the instrument, fearing injury to the womb; and that after re-introducing them, and while delivering, he had really to restrain the head from making too rapid progress through the soft parts; made no traction whatever; said the forceps seemed to stimulate the uterus to action. Both mother and child did well.

Dr. McRae mentioned a case of rigid os, where the head had caught and held the anterior lip immediately against the pubic bone for such a long time that the lip was immensely tumefied, pains were very severe; gave chloroform, ruptured the membrane; the head (very large) was forced down, and increased the pressure at each pain. By pushing up the uterus during the intervals of pain, he at last succeeded in getting the head in the proper axis of the os, and eventually slipped the os back over the head, introduced the forceps, and delivered; had but slight slitting of the frochette; used antiseptics freely to external parts; used no uterine or vaginal douche; the child was a remarkably large one; the case progressed favorably.

Dr. Noble suggested morphia and fld. ext. of black cohosh in cases of rigid os, having found great benefit from the combination. Said, also, that he was always in the habit of holding up the uterus until the head had properly and very thoroughly engaged the os.

Dr. Smith recommended the use of carbonic acid gas in cases of rigid os during labor. Dr. Smith said, a simple apparatus, consisting of a wide-mouth bottle, with a close-fitting cork, and a rubber tube two feet in length, one end of which was to be passed through the cork, the other end free (to be placed into the vagina, to within an inch or so of the os uteri) was all that was necessary. Into the bottle, one-third full of water, put the contents of the blue papers of two seidlitz powders, then dissolve in another vessel the contents of the two white papers; when ready for use, pour this solution into the bottle, cork tightly, and pass the free end of the tube into the vagina almost up to the os. The gas, he said, acted promptly in relieving the rigidity, and that it was a favorite remedy with the Germans.

Dr. Hardon said he had had a number of cases where there was rigidity of the os, with the anterior lip caught up behind the pubes. He said that this was caused by the head engaging at the brim, and displacing the waters, which should naturally come first, and assist in dilating the os. His plan was to push up the head, and allow the waters to come down, and perform their part; had had no trouble after thus adjusting matters.

Dr. Smith remarked that the genupectoral position would assist in carrying the head back.

Dr. Cooper gave an amusing account of his experience of the genupectoral position during labor. He said when he first graduated, he had a case of prolapsed cord, and placed the patient in the genupectoral position to aid him in reducing it, but could not keep her in the position; for as soon as a pain would come on, she would groan and throw herself on her side, and roll around; he would try it again, but as soon as a pain would begin, down she would go again. Said he tried to hold her up, but to no purpose; it would be over, and under, and roundabout every time, until at last, exhausted, he gave it up, and resorted to forceps, delivered quickly, and ended the struggle. Said he would defy any man to keep a woman, having labor pains, in any such position as the genupectoral. It was impossible.

